# Functional Hydrogels for Wearable and Implantable Neural Interfaces: Materials, Properties, and Applications

**DOI:** 10.3390/gels12100875

**Published:** 2026-09-28

**Authors:** Joowan Kim, Jaewoo Park, Yeojin Lee, Goeun Kim, Yunsu Shin, Seoyun Eom, Jinmo Jeong

**Affiliations:** 1 Department of Artificial Intelligence and Biomedical Engineering, The Catholic University of Korea, Bucheon-si 14662, Gyeonggi-do, Republic of Korea; joowan@catholic.ac.kr (J.K.);; 2 Department of Biomedical Software Engineering, The Catholic University of Korea, Bucheon-si 14662, Gyeonggi-do, Republic of Korea

**Keywords:** functional hydrogel, neural interface, wearable bioelectronics, implantable electrode, biocompatibility, tissue adhesion, human-machine interface

## Abstract

Functional hydrogels are increasingly used in neural interfaces. As hydrophilic polymer networks that combine tissue-like softness with tunable conductivity and adhesion, they relieve two fundamental limitations of rigid conventional electrodes within a single material: the mechanical mismatch that drives the chronic foreign body response, and the electrical mismatch that raises interfacial impedance. This review discusses functional hydrogels through their core interfacial properties of biocompatibility, adhesion, and interfacial impedance/conductivity, and highlights how these requirements differ between skin-mounted wearable and implantable devices. Their applications in neural recording, electrical and ultrasound neuromodulation, and external and robotic device control are then covered in detail. Finally, the key challenges and future opportunities are summarized, including gelation control, long-term in vivo stability, integration with robotic hardware, and clinical translation. As the field advances, functional hydrogels are poised to become a core platform for next-generation neural interfaces.

## 1. Introduction

Neural interfaces, devices that record the electrical activity of the nervous system and deliver stimulation to it, underpin a range of next-generation medical technologies, including brain–machine interfaces (BMIs), neuroprosthetics, and closed-loop neuromodulation therapies [1,2,3]. For such devices to operate reliably over the long term, the electrode must remain in intimate contact with the tissue to exchange signals. Conventional metal- and silicon-based electrodes, however, differ fundamentally from biological tissue in their physical properties, and this gives rise to serious problems at the electrode–tissue interface. This incompatibility can be divided into a mechanical and an electrical aspect. First, the mechanical incompatibility, the modulus mismatch, stems from the difference in stiffness. Neural tissue has an elastic modulus on the order of hundreds of pascals to a few kilopascals, making it very soft, whereas metal and silicon electrodes are on the order of gigapascals roughly 10^6^–10^9^ times stiffer than tissue [2,4,5]. Because tissue is in constant micromotion from respiration, heartbeat, and everyday movement, the junction between two materials of such disparate stiffness concentrates continuous stress, particularly shear stress, at the interface. This repeated mechanical insult provokes acute inflammation and ultimately a chronic foreign body response (FBR), macrophage-mediated inflammation together with, in the central nervous system, glial scarring driven by activated astrocytes and microglia, and the formation of a fibrous capsule. The outcome is progressive electrical insulation of the electrode and a steady decline in signal quality over time [6,7,8]. Second, the electrical incompatibility, the charge-carrier mismatch, arises from the different species that carry charge. Metal electrodes conduct charge through electrons, whereas tissue transmits signals through ions in the extracellular fluid. When electrons and ions cannot exchange charge smoothly at the electrode surface, the interfacial impedance becomes high. This demands higher voltages for stimulation, raising the risk of tissue damage, and lowers the signal-to-noise ratio (SNR) of recordings, degrading both the safety and the performance of the interface [9,10].

Functional hydrogels have emerged as an ideal alternative that can resolve both the mechanical and the electrical mismatch within a single material [11,12]. A hydrogel is a hydrophilic polymer network swollen with a large amount of water; it is about as soft as biological tissue, and its wet surface closely resembles the microenvironment of the extracellular matrix (ECM) [11]. Other soft materials, such as elastomers (e.g., polydimethylsiloxane (PDMS) or Ecoflex) and solid conducting polymers, have also been used to build soft neural interfaces, but each has clear limitations. Elastomers can be tuned to match the mechanical properties of tissue, yet they are inherently hydrophobic and insulating, making smooth charge transfer with ion-based tissue difficult. Indeed, the finding that a hydrophobic elastomer provokes a far stronger foreign body response than a hydrophilic hydrogel of the same modulus indicates that a water-bearing, hydrophilic surface—not mechanical softness alone—is a key factor for biocompatibility [13]. Conversely, conducting polymers or conductive fillers on their own offer excellent electrical properties but cannot simultaneously provide tissue-like softness and wetness [14]. A hydrogel, by contrast, can freely integrate additional functions such as conductivity and adhesion onto a soft, water-bearing backbone; its greatest distinction is the ability to reconcile the combined demands of softness, wetness, conductivity, and adhesion within a single material platform [14,15]. The “functional hydrogels” addressed in this review are precisely such smart materials, in which advanced functions, conductivity, tissue adhesion, and stimuli responsiveness, are designed directly into the polymer network.

The properties of a functional hydrogel are determined largely by two axes: the crosslinking method that forms the network, and the conduction mechanism that carries charge. First, crosslinking is divided into physical and chemical types, and the material’s macroscopic properties depend heavily on which is used. Physical crosslinking forms the network through reversible, non-covalent bonds, hydrogen bonding, ionic and electrostatic attraction, hydrophobic interaction, and chain entanglement that can break and re-form with the environment. This provides excellent self-healing and allows the material to be injected as a liquid and then gelled, but the relatively weak bonding results in low mechanical strength. Chemical crosslinking, by contrast, links the polymer chains permanently through covalent bonds, forming a far more robust and shape-stable network at the cost of reversible deformation. In practice, the two are therefore often combined to balance the trade-off between strength and reversibility, and such strategies have recently been extended to diverse form factors, including fibers [14,16]. Second, conductivity is imparted through two broad routes: ionic and electronic conduction. Ionic conduction, in which free ions move through the hydrated polymer network, synchronizes most naturally with the signaling scheme of biological tissue, but its absolute conductivity is relatively low and its performance can decline as water evaporates or ions leach out in the body [17,18]. Electronic conduction, which compensates for this, divides into two sub-strategies. One is a composite approach that disperses conductive nanomaterials (carbon nanotubes (CNTs), graphene, MXene, and the like) within an insulating hydrogel to form continuous electrical pathways (a percolation network). Here, however, reaching sufficient conductivity requires a high filler content that stiffens the material; so, a balance with flexibility is essential. The other uses an intrinsically conducting polymer (e.g., poly(3,4-ethylenedioxythiophene):poly(styrene sulfonate) (PEDOT:PSS)) as the matrix. In this case, electrons flow along the polymer backbone while ions migrate through the hydrated phase, enabling mixed ionic–electronic conduction. This dual-conduction character mediates between the ionic signals of tissue and electronic devices in a near-ideal way, achieving both remarkably low interfacial impedance and high charge-storage capacity, and is therefore attracting particular attention [19,20,21].

Although the material engineering strategies behind hydrogels are shared, the way they are best realized in a neural interface differs markedly with the application setting, that is, whether the device is a wearable one attached to the skin surface or an implantable one in direct contact with internal neural tissue. This review, by contrast, is distinguished by comparing the three core performance metrics of neural interface biocompatibility, interfacial adhesion, and interfacial impedance and conductivity across both wearable and implantable settings in an integrated way, and by spanning applications from biosignal recording and neuromodulation to the control of external devices and robots. This multifaceted analysis surfaces an engineering insight that mere enumeration of individual studies cannot capture: that the conformal-contact strategy of injecting a liquid precursor that fills every microscopic contour and gap of the tissue and then gels in place is a decisive factor governing interfacial performance beyond the choice of conduction mechanism itself [15]. From this perspective, this review examines in depth how the key properties of hydrogels are designed for wearable versus implantable settings, follows through concrete cases how these translate into real applications in biosignal recording, neuromodulation, and device control, and finally discusses the remaining challenges and future outlook for this material to advance beyond laboratory-scale demonstrations into a core platform for next-generation neural interfaces and bioelectronic robotic systems.

## 2. Critical Parameters of Hydrogels for Neural Interfacing

### 2.1. Biocompatibility

Biocompatibility refers to the ability of a material to perform its intended function reliably, in contact with biological tissue or skin, without eliciting harmful adverse effects [22]. Wearable and implantable applications differ markedly in the immunological mechanisms and time scales that define this requirement. In wearable devices, the central concern is preventing localized irritant and allergic contact dermatitis, whereas in implantable devices, the key challenge is controlling the systemic foreign body response (FBR) cascade that proceeds through protein adsorption, acute inflammation, chronic inflammation, and fibrous encapsulation [23]. Hydrogels possess a high water content, a soft, tissue-like consistency, and a hydrophilic surface, allowing them to simultaneously mitigate interfacial energy mismatch and mechanical mismatch with skin and biological tissue, which gives them a strong advantage in both application domains [24]. In particular, the severe modulus mismatch between neural tissue (1–100 kPa) and conventional metal or silicon electrodes (10–100 GPa) has been identified as a major cause of chronic shear stress at the interface during micromotion, driving microglial activation and glial scar formation [1,8]. However, mechanical modulus matching is a necessary but not sufficient condition for biocompatibility. Design variables such as the chemical composition of the material, the crosslinked network structure, and surface properties determine minimized skin irritation and safe adhesion in wearable applications, and modulus-independent suppression of the foreign body response in implantable applications.

#### 2.1.1. Wearable—Skin Irritation & Adhesion Safety

The biocompatibility of skin-attached hydrogels is primarily determined by the chemical purity and composition of the material. Wong et al. used gas chromatography to quantitatively show that unreacted acrylate-based crosslinker pentaerythritol triacrylate (PETRA) in UV-crosslinked poly(ethylene oxide) (PEO) hydrogels can act as a potent skin irritant and allergen, and confirmed that sufficiently complete polymerization minimizes this residue and eliminates the irritant potential [25]. Natural polymers such as silk fibroin, gelatin, cellulose, chitosan, and alginate are relatively free of such residual-chemical concerns. Lee et al. reported that a genipin-crosslinked gelatin/PEDOT:PSS electrode achieved an adhesive strength of 0.85 N while dramatically reducing skin damage compared to commercial electrodes with a chemical adhesive layer [26]. Synthetic polymers achieve low modulus and strong skin adhesion simultaneously through control of crosslink density and plasticizer content. Feig et al. controlled the crosslink density of an interpenetrating network (IPN) to tune the elastic modulus across a range of 8–374 kPa (three orders of magnitude) without any loss of conductivity [27]. Soni et al. optimized the crosslinker (MBAA) concentration in a PEDOT:PSS/polyacrylamide (PAAm) hydrogel to improve skin adhesion and recorded electrocardiography (ECG) signals with 99.6% agreement with a commercial electrode [28]. Liu et al. used a plasticizer to relax the crosslinking of a PAA network, simultaneously achieving a strong adhesion of 171 kPa on poly(ethylene terephthalate) (PET), sub-1 kΩ interfacial impedance, and antibacterial activity, even at an ultrasoft modulus of 0.76 kPa [29]. The AMPS-based AIRTrode developed by Hsieh et al. maintained an adhesive strength of 0.92 ± 0.21 N/cm even after more than 20 repeated attachment–detachment cycles, demonstrating reusability without skin damage [30]. The biocompatibility of such wearable hydrogels is conventionally verified through 3-(4,5-dimethylthiazol-2-yl)-2,5-diphenyltetrazolium bromide (MTT) cytotoxicity assays and skin patch tests; the antibacterial semi-dry electroencephalography (EEG) electrode developed by Wang et al. likewise underwent this verification and confirmed long-term, repeatable-use safety [31].

Chemical purity is itself a function of how completely the reaction proceeds, and for natural polymers, a class that includes silk fibroin, used in both skin-contacting and implanted neural devices, that completeness is governed by chain conformation rather than by the chemistry alone. Exposing silk nanofiber solutions held in different conformational states to identical reaction conditions, Hang et al. found that amorphous nanofiber approached the theoretical grafting limit set by tyrosine content in diazonium coupling to phenol groups, whereas β-sheet-rich nanofiber converted the least. β-sheet formation moves phenol groups inward, while carboxylic groups migrate outward; so, EDC/NHS coupling responds in the opposite direction, and amorphous solutions, which do not drift in aggregate size, were identified on this basis as the better starting point for controlled modification [32]. For a skin-contacting material, the consequence is reproducibility: the degree of substitution sets the surface chemistry the skin meets; so, safety established for one batch does not transfer unless the conformational state is controlled. The same control can also be used constructively since modifying the amorphous state, inducing the β-sheet transition and modifying again yields a dual-functionalized material without blocking and deblocking [32]. The consequences for electrical performance are taken up in Section 2.3.2.

#### 2.1.2. Implantable—Immune Response & Surface Chemistry

For implantable hydrogels, material and crosslink structure design determine the success of FBR control. FBR mitigation is commonly understood to depend primarily on modulus matching between the tissue and the electrode [1,8], but the porous network and hydrophilic surface properties of hydrogels intervene directly in the early immune response independently of modulus. Capuani et al. and Davenport Huyer et al. confirmed that hydrophobic surfaces polarize macrophages toward an inflammatory (M1) phenotype, whereas hydrophilic surfaces and controlled porous structures induce an anti-inflammatory (M2) polarization [23,33]. Jeong et al. coated a sciatic nerve electrode with a gelatin hydrogel and with Ecoflex silicone, each possessing an equivalent modulus (~80 kPa), and compared the in vivo response. Despite identical mechanical properties, the Ecoflex-coated group showed fibrosis three times that of the sham group and CD68 expression up to 23.7 times higher, whereas the gelatin hydrogel group showed anti-inflammatory characteristics with no significant difference from the sham group even after 15 days [13]. This result reflects the fact that adjusting crosslink density (the gelatin-to-genipin ratio) tuned the modulus across a range of 24–792 kPa while preserving the intrinsic immunomodulatory function of the hydrophilic polymer network. The local surface chemistry of a material is also a major variable determining FBR. Vegas et al. built a library of alginate-based hydrogels and implanted them in primates, finding that only a small subset of candidates chemically modified with triazole derivatives markedly suppressed FBR and capsule formation for more than 6 months [34]. This effect was determined not by physical properties such as protein adsorption or bulk modulus, but by the local distribution of surface chemistry. Ionically crosslinked (Ca^2+^-based) hydrogels such as alginate have the limitation that long-term structural stability can degrade as ions leach out in the physiological fluid environment, but they also carry the intrinsic advantage that this same in vivo self-absorption and degradation process can secure excellent biocompatibility with the surrounding tissue.

A further determinant of chronic performance that is specific to hydrogels is swelling. Because a hydrogel equilibrates with the surrounding fluid, its dimensions and water content continue to change long after implantation: volumetric expansion presses the device against the surrounding parenchyma and sustains the mechanical insult that drives the foreign body response, while the accompanying change in polymer volume fraction alters ion mobility and interfacial contact so that the impedance measured at implantation is not the impedance at which the device later operates. Three distinct responses have emerged. The first blocks water uptake physically: an atomic-layer-deposited SiO2 layer on crosslinked poly(vinyl alcohol) hydrogel fibers prevented swelling under accelerated stability testing and preserved the mechanical and optical properties that uncoated fibers lost, allowing them to remain functional as waveguides for optogenetic stimulation in vivo [35]. The second makes the interface withstand the volume change rather than prevent it; in conducting-polymer coatings, the swelling is electrochemically driven, and covalently anchoring the polymer chains into a PEDOT-based interpenetrating network raised the interfacial toughness enough that electrodes in freely moving mice held 100–250 kΩ at 1 kHz across four weeks of recording and stimulation [36]. The third exploits the volume change instead: a nanofibrous thermoresponsive network of a poly(N-isopropylacrylamide)-based copolymer and a polythiophene contracts at body temperature and becomes more conductive as it does so, and probes coated this way recorded deep-brain activity over six weeks with diminished reactive astrogliosis [37]. What these routes share is that swelling is treated as a design variable rather than a nuisance, yet the swelling ratio and its time course are still rarely reported alongside modulus and impedance.

In summary, wearable hydrogels optimize crosslink density and material selection for minimizing skin irritation and ensuring repeated-adhesion safety, whereas implantable hydrogels apply the same variables toward porosity-, hydrophilicity-, and surface-chemistry-based immunomodulation (M2 polarization) and biocompatibility that operate independently of modulus matching.

### 2.2. Adhesion

Ensuring the long-term stability of the interface between neural tissue and electronic devices is a key determinant of the long-term reliability of neural interfaces. In particular, securing interfacial adhesion technology that can overcome the mechanical and biochemical mismatch between flexible hydrogel electrodes and biological tissue interfaces is essential. Hydrogel interfacial adhesion design is largely built around two chemical axes, non-covalent bonding and covalent bonding, with the required bonding characteristics and design strategy differing depending on the application environment (wearable vs. implantable).

#### 2.2.1. Non-Covalent Interfacial Bonding

Non-covalent bonding collectively refers to hydrogen bonding, ionic bonding, van der Waals forces, and mechanical interlocking, which operate through electrostatic attraction without electron-pair sharing [14]. Although individual bonds are relatively weak, their reversibility, readily dissociating and reforming in response to external stimuli such as temperature or pH, makes them preferred in applications requiring non-damaging attachment/detachment. Here, the water content of the hydrogel acts as an important variable in interfacial adhesion. High water content increases chain flexibility and contact area, promoting hydrogen bond formation, but can reduce the mechanical strength of the hydrogel itself, leading to interfacial slippage. Conversely, lower water content improves the structural stability of the matrix but weakens initial adhesion due to reduced interfacial flexibility a trade-off that always exists.

##### Wearable

In wearable applications, the hydroxyl, carboxyl, and amino groups of hydrogels form hydrogen bonds with the skin surface, and the thinner and more flexible the hydrogel, the higher its conformability to fine skin contours, simultaneously enhancing contact area and van der Waals forces. A 392 nm thick ultrathin organohydrogel electrode achieved a high adhesion strength of 365.8 µJ/cm^2^ through this principle alone without chemical bonding. It maintained adhesion performance after 1000 cycles of deformation at 100% strain and stably recorded 9 consecutive days of electrocardiogram (ECG) (Figure 1A) [38]. A temperature-responsive hydrogen-bonding design follows the same principle. A poly(vinyl alcohol)/phytic acid/gelatin hydrogel utilizes the temperature reversibility of multiple hydrogen bonds to adhere strongly with a hot compress and detach easily without skin irritation or pain with a cold compress [39].

##### Implantable

Interfaces composed solely of non-covalent bonding have a fundamental limitation. Their adhesion strength, based on physical adsorption, is inherently weak and vulnerable to fatigue. Overcoming this requires the simultaneous design of a dissipative hydrogel matrix and physical anchoring [40]. Nevertheless, poly(ethylene glycol) dimethacrylate (PEG-DMA)-coated neural probes significantly reduced local stress caused by micromotion and glial scar formation, demonstrating that a mechanically matched hydrogel layer can buffer micromotion at the probe–tissue interface [5].

#### 2.2.2. Covalent Interfacial Bonding

Covalent bonding is further divided, depending on whether it can re-form, into static covalent bonds (carbon–carbon, silicon–oxygen, amide bonds, etc., which are irreversible) and dynamic covalent bonds (imine, disulfide, boronic ester bonds, etc., which are reversible) [14]. Forming them on tissue is not straightforward, because the surface is hydrated and the only abundant reactive groups are the amines and thiols of surface proteins; the hydrogel must therefore carry complementary groups such as catechol-derived quinones, NHS esters, or aldehydes, and must displace interfacial water before bonding [11]. Strength is not set by the bond alone: covalent anchoring raises the intrinsic work of adhesion, but a tough interface also requires the hydrogel network itself to dissipate energy during detachment [41]. Whether the bond can re-form then decides the trade-off, as static bonds give permanence but preclude non-damaging removal, whereas dynamic bonds exchange fast enough to self-heal, with boronic ester exchange restoring a broken network within 5 s owing to its low activation energy (≈20 kJ/mol) [42]. Implanted devices must hold for months against continuous micromotion and are never meant to be peeled off; so, static covalent bonding dominates there, whereas wearables, attached and removed repeatedly, rely on dynamic covalent and non-covalent bonding.

##### Wearable

Catechol (dopamine) chemistry represents the covalent adhesion strategy for wearables. The quinone generated by oxidation of dopamine forms covalent bonds with amine and thiol groups on the skin surface. An electrode patch based on polydopamine/silver-platinum nanowires (PDA-Ag@Pt NW) applying this chemistry recorded ECG, electromyography (EMG), and EEG with better resolution than commercial Ag/AgCl electrodes, showing a low skin-contact impedance of 6.7 kΩ at 1 kHz and a self-healing efficiency of 75–95% [43]. However, since the quinone-amine bond is close to a static covalent bond that does not re-form, the trade-off between adhesion strength and reusability must be considered for wearable applications requiring repeated attachment and detachment.

##### Implantable

Chemical covalent bonding is practically essential for implantables, but a challenge remains in simultaneously satisfying the conflicting requirements of adhesion permanence and repositionability during a procedure. To address this, a hydrogel tape was proposed in which catechol (dopa) chemistry is precisely controlled via electro-oxidation. Within the first few seconds, instant adhesion is achieved through non-covalent bonds such as ionic interactions, cation–π interactions, and hydrogen bonding. Over the following hours, dopaquinone gradually reacts with amine groups on the tissue surface to form covalent bonds, strengthening the adhesion energy to approximately 1268 J/m^2^ through this time-dependent mechanism (Figure 1B). This approach was validated in actual implantable applications, such as attaching an electronic device to a beating rabbit heart, and it showed the advantage of retaining most of its initial adhesion strength even after being repositioned multiple times during a procedure [44].

A similar time-dependent cooperative principle has also been applied to actual cardiac patch materials. A mussel-inspired adhesive Janus hydrogel, implanted at the site of myocardial infarction, promoted the maturation and function of cardiomyocytes, reduced oxidative damage and inflammatory response, and reconstructed electrical conduction and blood flow. Its adhesion strength increased from 251 kPa to 16,101 kPa one month after implantation (Figure 1C) [45]. In this material, the catechol group of caffeic acid, the carboxyl group of acrylic acid, and the amine group of polyethylenimine first adhere to the tissue via non-covalent bonds. Afterward, the oxidized catechol (quinone) and aldehyde groups react with amine groups on the tissue to form covalent bonds, showing a time-dependent cooperative structure in which adhesion is progressively strengthened. Notably, this material was designed to allow for non-damaging detachment upon glutathione (GSH) stimulation, representing a case in which the robustness of static covalent bonding and the reactivity of dynamic covalent bonding are introduced together.

In summary, wearable neural interfaces are recommended to adopt a design based on non-covalent and/or dynamic covalent bonding that allows for gentle attachment and detachment, whereas implantable neural interfaces are recommended to adopt a design based on static covalent bonding, or a dual mechanism in which initial non-covalent bonding and subsequent covalent bonding cooperate in a time-dependent manner.

**Figure 1:**
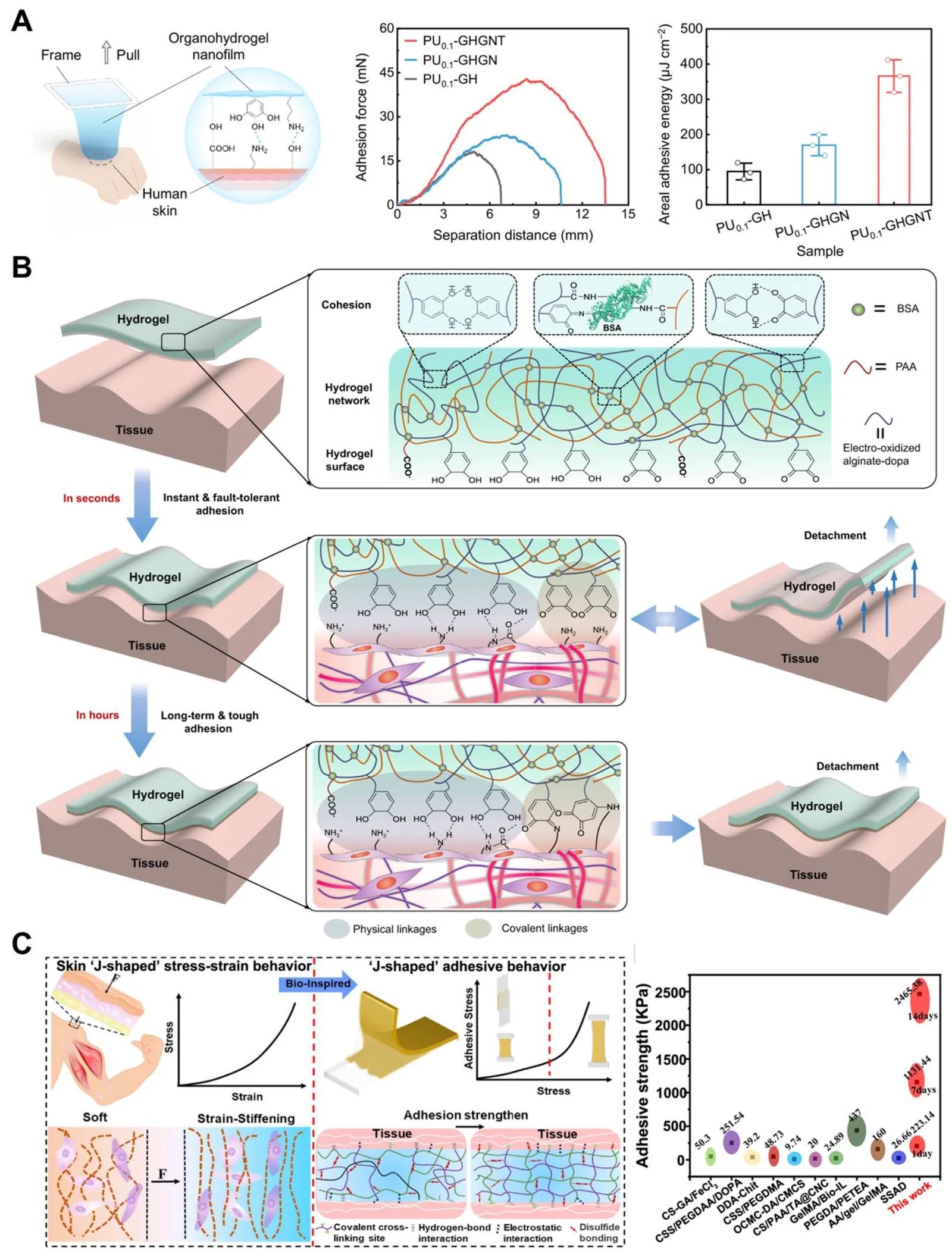
Spectrum of hydrogel adhesion strategies for wearable and implantable neural interfaces. (**A**) Non-covalent, hydrogen-bond-mediated adhesion of an organohydrogel nanofilm to skin, achieved without chemical bonding. Adapted from Wang, Z. et al. Nat. Commun. 2025, 16, 10103, under a CC BY-NC-ND 4.0 license © 2025 The Author(s) [38]. (**B**) Time-dependent adhesion mechanism of the Electro-Ox hydrogel tape, combining rapid non-covalent interactions with gradual dopaquinone–amine covalent bond formation over hours. The downward arrows indicate the transition from rapid adhesion within seconds to stronger long-term adhesion over hours, while the horizontal arrows indicate the corresponding change in detachability. Adapted from Xue, B. et al. Nat. Commun. 2021, 12, 7156, under a CC BY 4.0 license [44]. (**C**) Adhesion-adaptive stiffening behavior observed as this non-covalent-to-covalent transition progresses in an implanted Janus cardiac patch hydrogel. Adapted from He, Y. et al. Nat. Commun. 2022, 13, 7666, under a CC BY 4.0 license [45].

### 2.3. Interfacial Impedance and Conductivity

For a neural interface to reliably support both recording and stimulation, efficient charge transfer across the electrode–tissue interface is essential, in addition to mechanical and biochemical compatibility. Biological tissue conducts current ionically, through the migration of Na^+^, K^+^, and Cl^−^ ions in the extracellular fluid, whereas conventional electrode materials, such as metals, silicon, and most conductive fillers, conduct electronically, through free electrons [11]. This mismatch in charge-carrier type, compounded by the modulus mismatch discussed in Section 2.1, gives rise to a high-impedance electrochemical double layer at the interface [46], which attenuates recorded biopotential signals and raises the voltage threshold required for safe charge injection during stimulation [9,10,11]. This problem can be addressed along two axes: physically lowering impedance through electrode geometry and interfacial contact design [47], and chemically raising conductivity through the hydrogel’s own conduction mechanism [19].

#### 2.3.1. Interfacial Impedance

The electrode–tissue interface is commonly modeled as an electrical equivalent circuit (Figure 2A) [48]. This circuit combines the resistance of the electrolyte (tissue), the capacitance of the electrical double layer formed at the electrode surface, and the charge-transfer resistance, and impedance is jointly determined by these capacitive and resistive components [15,48]. Because the capacitive component is proportional to the effective contact area between electrode and tissue, electrode thickness and surface area are primary geometric variables governing impedance [15]; that is, a thicker electrode lengthens the current path and increases resistance, while a smaller contact area with tissue reduces capacitance and raises impedance. Another factor determining the quality of interfacial contact is the microscopic air gap that remains between electrode and tissue [48,49]. Goyal et al. modeled this gap as a thin dielectric layer and represented the electrode–skin contact as a parallel-plate capacitor (Figure 2B) [49]. In this model, the contact capacitance (C_c_) is given by C_c_ = ε_0_·ε_r_·A/d, where A is the contact area and d is the effective distance between electrode and skin, i.e., the gap thickness [49,50]. This expression quantitatively shows that even a small reduction in gap thickness markedly increases capacitance and thus lowers impedance.

Ultimately, the practical key to lowering interfacial impedance lies in whether the hydrogel can conform to the fine irregularities, curvature, and gaps of the tissue surface without leaving an air gap [47,48].

#### 2.3.2. Conductivity

The intrinsic ionic conductivity of biological tissue is on the order of 3 × 10^−3^–7 × 10^−3^ S/cm (0.3–0.7 S/m) [17], and the mechanisms by which hydrogels achieve conductivity at or above this level fall broadly into (1) ionic conduction and (2) electronic or mixed ionic–electronic conduction.

(1) Ionically conductive hydrogels: The mesh size of a hydrogel network is on the order of 5–50 nm depending on crosslinking conditions, far larger than a water molecule, allowing ions to move relatively freely within the hydrated matrix (Figure 2C) [51,52]. The ionic conductivity (σ) of a hydrogel is described by σ = Σ_i_ n_i_·μ_i_·Z_i_·e (where n_i_ is the carrier concentration, μ_i_ the ionic mobility, Z_i_ the ion valence, and e the elementary charge), and is therefore governed mainly by ion concentration and mobility [18]. Two representative fabrication strategies are used for ionically conductive hydrogels. The first is the immersion method, in which a polymer matrix such as poly(vinyl alcohol) (PVA), PEG, or a polyacrylamide–alginate (PAAm–alginate) blend is first crosslinked and then immersed in an ionic solution containing Ca^2+^, Na^+^, Al^3+^, or Cl^−^**,** allowing ions to diffuse into the matrix along the concentration gradient [52,53,54]. Because the pre-crosslinked matrix does not obstruct ion pathways, the conductivity of this route is governed mainly by the ion species and the concentration of the immersion bath. Reconstructed alginate hydrogels loaded with Li^+^ reach 2.2 × 10^−3^ S/cm when crosslinked with Ca^2+^/Fe^3+^ and 7.8 × 10^−3^ S/cm when crosslinked with Al^3+^, and lose their ionic conductivity entirely once the mobile ions are removed in pure water [52]; hydroxypropyl cellulose (HPC)/PVA hydrogels soaked in 5 m NaCl reach 1.7 × 10^−2^–3.4 × 10^−2^ S/cm as the HPC content increases from 0 to 3.75 wt% [53]; and a cellulose–bentonite hydrogel soaked in 2 M LiCl reaches 8.99 × 10^−2^ S/cm, approximately 90-fold higher than the same network without LiCl (≈1 × 10^−3^ S/cm) [55]. The second is direct mixing, in which a concentrated salt (e.g., LiCl) or an ionic liquid is blended directly into the hydrogel precursor [56,57], and combining ionic liquids with crosslinkers under appropriate encapsulation can further improve functionality and stability [58]. In a representative example, introducing 5 wt% LiCl into an alginate–polyacrylamide network lowers its intrinsic impedance from the MΩ range to 270 Ω at 1 Hz [56]. Such ionic doping can raise the conductivity from roughly 10^−5^ S/cm to 10^0^ S/cm [59,60,61], but in vivo application requires attention to an upper concentration limit: physiological ion concentrations are typically below 300 mM, and doping in excess of 1 M can establish a steep concentration gradient between the hydrogel and the surrounding tissue. This can drive ion efflux into the tissue after implantation, degrading the electrode’s long-term electrical stability and disturbing the osmotic balance of the surrounding biological environment.
(2) Electronically and mixed conductive hydrogels: Electronic conduction arises from the delocalized π-electrons that move along the backbone of conjugated polymers such as PEDOT:PSS, polypyrrole (PPy), and polyaniline (PANI) (Figure 2D) [11,19]. Conducting-polymer-based hydrogels typically exhibit conductivities of 10^0^–10^2^ S/cm and Young’s moduli spanning 100 kPa to 1 GPa [11,62]. Several microstructural strategies have been reported to further raise this electronic conductivity. Tuning the ratio of PEDOT:PSS to hydrophilic polyurethane to form a bicontinuous phase morphology can achieve conductivities above 11 S/cm [20], while template-directed assembly—growing ultrathin PEDOT:PSS fiber networks along poly(acrylic acid) (PAA) chains—can reach 247 S/cm [63]. Treating the material with sulfuric acid or a strongly ionic liquid to break the electrostatic association between positively charged PEDOT and negatively charged PSS, thereby reorganizing the PEDOT chains [64], strengthens π–π interactions and allows ionic-liquid-processed PEDOT:PSS hydrogels to reach conductivities of 47.4 S/cm [65]; laser-induced phase separation, which forms a PEDOT-rich continuous phase, can raise this further to 670 S/cm [62]. Beyond these conducting-polymer routes, a purely filler-based composite of micrometer-sized silver (Ag) flakes percolating in a PAAm–alginate hydrogel reaches 374 S/cm [66].
(3) Filler-based nanocomposite hydrogels: Incorporating carbon-based fillers (CNTs, graphene), metal nanoparticles, or MXenes into an otherwise insulating hydrogel matrix is another widely studied approach. The hydrogel becomes conductive only once physical contact between fillers exceeds the percolation threshold and forms a continuous electron-transport pathway; this threshold depends strongly on the filler aspect ratio, dispersion, aggregation tendency, and surface modification. In protein matrices relevant to nerve-contacting devices, this dispersion can also be templated by the host itself, as in silk-fibroin nanofibers co-assembled with graphene sheets under an electric field, where the conductive filler is carried alongside aligned topography and a compressive modulus of 0.2–0.8 kPa as co-presented cues for nerve cells [67]. Huang et al. incorporated high-aspect-ratio CNTs into a semicrystalline PVA hydrogel and applied repeated cyclic stretching (20% strain, up to 5000 cycles) to realign the CNT bundles along the stretching direction, reconstructing an electrically anisotropic percolation pathway (Figure 2E) [68]. This yielded anisotropic hydrogel fibers whose impedance decreased selectively along the stretching direction (33.20 ± 9.27 kΩ at 1 kHz), which were implanted into mouse spinal cord and muscle to enable stable recording of electromyographic and spinal neural signals for over eight months alongside optogenetic stimulation [68]. This demonstrates that, rather than randomly dispersing fillers, actively controlling filler alignment through mechanical deformation can achieve a low-impedance percolation network. A genetically encoded route to the same end has also been demonstrated, in which a graphene-oxide-binding domain built into a silk-elastin-like protein organizes the filler and thermally driven contraction of the gel brings the bound domains into contact, switching conductivity reversibly by about an order of magnitude to 5 × 10^−3^ S/cm [69]. Filler-based hydrogels of this kind are generally reported to span conductivities of 10^−5^–10^2^ S/cm and Young’s moduli of 1 kPa–10 MPa [11,70]. However, such filler-based approaches remain vulnerable to loss of conductivity from localized filler agglomeration, which breaks conductive pathways, or from filler leaching out of the polymer network under repeated stretching or fatigue; ensuring uniform filler dispersion and robust interfacial bonding with the polymer chains therefore remains a central engineering challenge.

These three conduction strategies, (1) ionic conduction, (2) electronic/mixed conduction using a conducting polymer as the matrix itself, and (3) nanocomposite approaches that disperse fillers in an insulating matrix, differ in their representative materials, fabrication processes, and achievable conductivity ranges, providing a basis for selecting a strategy in neural interface design. Table 1 compares representative examples across these conduction types, showing at a glance how far conductivity can vary with material and fabrication choice, and which physical mechanism underlies each strategy. Which of the three strategies is appropriate is not decided by conductivity alone, because the highest reported values do not belong to the materials best matched to tissue. The advantages and limitations of each route are summarized in Table 1; what they imply for design is that the choice should follow the dominant requirement rather than the peak value. Where charge-injection density governs the outcome, a conducting-polymer matrix is warranted despite moduli that reach the megapascal-to-gigapascal range. Where chronic mechanical compliance governs it, an ionic or lightly filled network is preferable even at a substantially lower conductivity since the accessible range of roughly 10^−5^–10^0^ S/cm already overlaps that of tissue itself. Where the interface must survive large repeated deformation, filler alignment and interfacial bonding to the host polymer matter more than the conductivity quoted at rest, because percolation-based conduction fails by losing contact rather than by being intrinsically low. Two considerations cut across this comparison. Hybrid designs that pair a conducting-polymer phase with an ionically conductive network are increasingly used to avoid forcing the choice at all, and, as Section 2.3.1 showed, conformal contact can lower interfacial impedance by more than a change in conduction mechanism does; so, the geometry of contact is worth settling before the conduction strategy is optimized. The revised Table 1 also reports the charge-transport mode, network/crosslinking, representative mechanical properties, and characterization methods.

**Figure 2:**
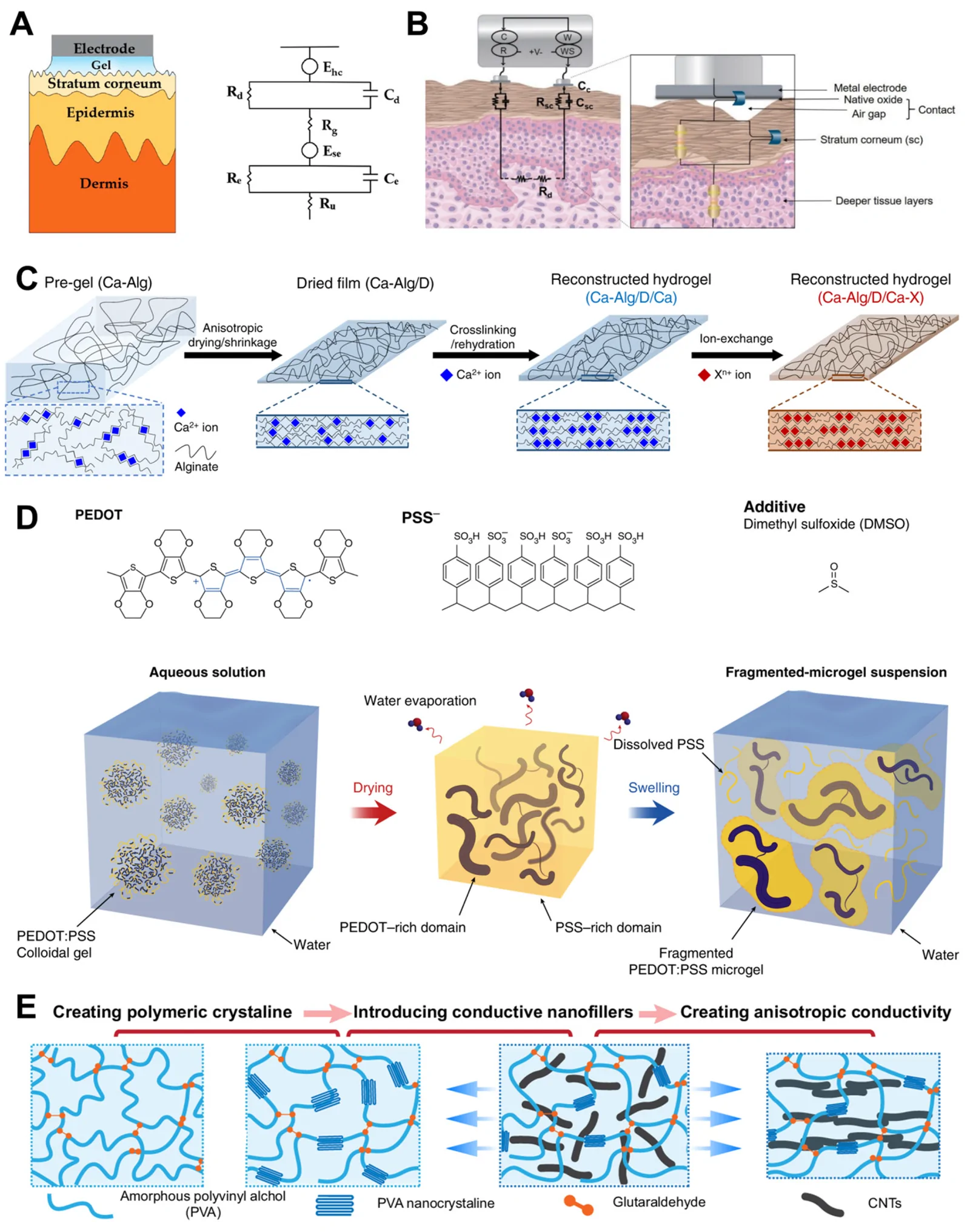
Mechanistic basis of interfacial impedance and conductivity in hydrogel neural interfaces. (**A**) Equivalent circuit model of the electrode–tissue interface, combining electrolyte resistance, double-layer capacitance, and charge-transfer resistance. Adapted from Fu, Y. et al. Sensors 2020, 20, 3651, under a CC BY 4.0 license [48]. (**B**) Parallel-plate capacitor model of the air gap between a dry electrode and skin, incorporating native-oxide and stratum-corneum contact resistance. Adapted from Goyal, K. et al. Sensors 2022, 22, 8510, under a CC BY 4.0 license [49]. (**C**) Reconstruction process for an ionically conductive alginate hydrogel via anisotropic drying/shrinkage, ionic crosslinking with rehydration, and subsequent ion exchange. Adapted from Ji, D. et al. Nat. Commun. 2022, 13, 3019, under a CC BY 4.0 license [52]. (**D**) Chemical structures of PEDOT, PSS, and dimethyl sulfoxide (DMSO), together with the typical drying and swelling processes of pristine PEDOT:PSS without DMSO. The red arrow indicates drying accompanied by water evaporation, whereas the blue arrow indicates subsequent swelling, leading to a fragmented PEDOT:PSS microgel suspension with dissolved PSS. Adapted from Lu, B. et al. Nat. Commun. 2019, 10, 1043, under a CC BY 4.0 license [19]. (**E**) Schematics of PVA hydrogel microstructures across sequential phases—chemical cross-linking, nanocrystalline growth, incorporation of CNTs, and cyclic-training-induced realignment of CNTs—yielding an anisotropic percolation network. Adapted from Huang, S. et al. Nat. Commun. 2025, 16, 1127, under a CC BY-NC-ND 4.0 license. © 2025 The Author(s) [68].

Interfacial impedance is thus governed jointly by a physical design axis, electrode geometry and interfacial contact, and a material-chemistry axis spanning ionic conduction, electronic conduction, and filler-based nanocomposites. How these properties translate into the recording and stimulation performance of actual wearable and implantable neural interfaces is addressed in Section 3 through specific application examples.

## 3. Applications of Hydrogels in Neural Interfacing

### 3.1. Neural Recording

#### 3.1.1. Wearable Biopotential Recording

In wearable hydrogels, recent work has increasingly shown that the process and morphological methodology used to deliver and conform the material to the skin surface has a more direct effect on lowering interfacial impedance than the choice of conduction mechanism itself. To fundamentally address irregular surfaces such as the hair-bearing scalp, delivering the hydrogel precursor to the skin in liquid form, injectable or paintable, and then gelling it in situ to conformally fill fine contours and gaps has become a clear recent trend. Hsieh et al. mixed PEDOT:PSS with the ionically conductive monomer AMPS (2-acrylamido-2-methylpropane sulfonic acid) in DMSO to develop an injectable hydrogel electrode (AIRTrode) that combines an electronic pathway (PEDOT:PSS) with an ionic pathway (the sulfonic acid groups of AMPS). By tuning the composition ratio, this precursor can be fabricated either as an on-site formed (injectable) electrode or as a pre-formed, self-contained electrode (Figure 3A). It spontaneously gels at room temperature through DMSO-induced phase separation among PEDOT:PSS chains, without any external crosslinker or stimulus, allowing it to be injected as a viscous liquid into the gaps between hairs and solidify in situ. The electrode maintained a low impedance of 17.53 kΩ over continuous recordings exceeding eight hours, achieved repeatable adhesion with a peel strength of 0.92 N/cm, and delivered an SNR of 23.97 dB versus 17.98 dB for a commercial wet electrode—enabling polysomnography-grade sleep-stage classification with high agreement to a commercial reference [30]. Wang, Y. et al. developed an injectable eutectogel electrode based on gelatin (Figure 3B). This hydrogel undergoes a temperature-dependent reversible phase transition, gelling rapidly in situ on the scalp from a liquid state to form a robust self-adhesive interface. It maintained mechanical durability over 1000 cycles at 100% strain, exhibited an adhesion strength of 0.7 N/cm on human epidermis (1.7 N/cm relative to a commercial Ag/AgCl electrode), and showed excellent anti-drying properties with negligible water loss over seven days, demonstrating its capability for high-quality, long-term scalp EEG monitoring [71]. Tang, H. et al. presented an in situ forming hydrogel electrode (ISF-HE) that establishes a highly conformal interface on curved biological surfaces without any auxiliary adhesive layer. This electrode had a compliance (≈0.02 kPa^−1^) comparable to that of skin, allowing it to move synchronously with skin deformation, and a fast response time of ≈180 ms that accurately tracked both large and small movements. By forming a non-gapped electrode–skin interface, it achieved an ultralow interfacial impedance of ≈50 kΩ—nearly an order of magnitude lower than commercial Ag/AgCl electrodes and other previously reported electrodes—translating into stable recording of biosignals even during outdoor exercise [72].

#### 3.1.2. Implantable Neural & Peripheral Electrophysiological Recording

Implantable neural interfaces face two challenges simultaneously: mechanical damage during insertion, and chronic interfacial degradation driven by the foreign body response (FBR) and micromotion after implantation. Hydrogels serve as a mediator that addresses both requirements at once, offering tissue-like modulus together with anti-fouling surface chemistry.

Park et al. integrated a multifunctional neural probe built from microscale fibers into a soft hydrogel matrix. This hydrogel hybrid probe was designed to minimize mechanical impact on brain tissue, and is fabricated by thermally drawing the functional fiber units and then directly polymerizing the hydrogel matrix within the fiber assembly (Figure 3C). The resulting hydrogel exhibits adaptive bending stiffness, with its elastic modulus changing by nearly three orders of magnitude between the dry and fully hydrated states: it remains relatively stiff for insertion, and then softens to brain-tissue-like compliance once hydrated in vivo, minimizing interfacial damage from micromotion. This enabled a long-term, stable multifunctional interface that simultaneously supports electrophysiological recording, optogenetic stimulation, and drug infusion [73].

Separately from this mechanical adaptation strategy, an approach that leverages the hydrogel’s surface chemistry itself to suppress the foreign body response of chronically implanted electrodes—while supporting both recording and stimulation—has also been reported. Jeong et al. developed a hybrid neural interfacing device (HNID) in which a nanogranular gold (Au) electrode is wrapped in a genipin-crosslinked gelatin hydrogel to conformally encircle the sciatic nerve. This suppressed inflammation around the sciatic nerve while achieving stable bidirectional neural communication—supporting both recording and stimulation—for over 15 days [13].

A similar design principle, achieving chronic bidirectional neural interfacing through a compressive-stress-free conformal interface, has also been observed in other implantable devices applied to the sciatic nerve. Song et al. developed an adaptive self-healing electronic epineurium (A-SEE) combining self-healable insulating and conducting materials, forming a compressive stress-free, strain-insensitive interface with the rat sciatic nerve without the need for sutures or adhesives (Figure 3D). This interface autonomously adapts to the dynamic expansion and contraction of the nerve, minimizing nerve-compression-induced immune responses and device failure, and thereby achieved stable bidirectional (recording and stimulation) neural signaling at the sciatic nerve over 14 weeks [74].

These examples together demonstrate that hydrogel-mediated, low-FBR implant interface strategies can be applied in common to both cortical recording and peripheral neural communication.

**Figure 3:**
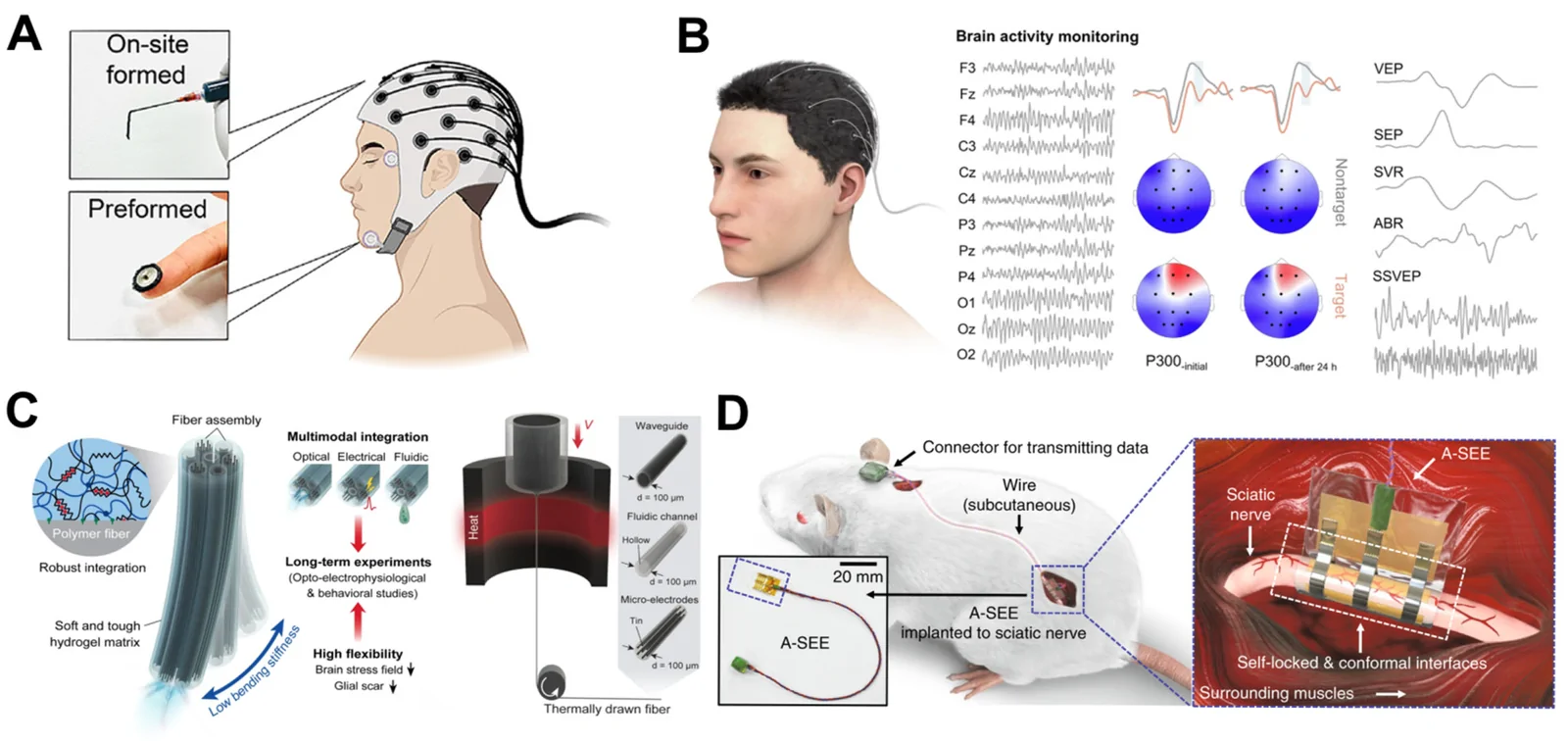
Representative wearable and implantable hydrogel interfaces for neural biopotential recording. (**A**) Pictures of the fabricated AIRTrode with different composition ratios to form an on-site formed (injectable) electrode or pre-formed (self-contained and preshaped) electrode. Adapted from Hsieh, J.-C. et al. Device 2024, 2(2), 100182, under a CC BY 4.0 license [30]. (**B**) Injectable eutectogel for high-quality, long-term brain activity recording. Adapted from Wang, Y. et al. npj Flex Electron 2025, 9, 125, under a CC BY-NC-ND 4.0 license. © 2025 The Author(s) [71]. (**C**) A conceptual illustration of the hydrogel hybrid probe design and its application to minimize impact on brain tissue. Fabrication of the hydrogel hybrid probe including thermal drawing of the functional fiber units. Adapted from Park, S. et al. Nat Commun 2021, 12, 3435, under a CC BY 4.0 license [73]. (**D**) Schematic of an adaptive self-healing electronic epineurium (A-SEE) interfaced with a rat sciatic nerve, illustrating a compressive stress-free, chronic bidirectional neural interface design. Adapted from Song, K.-I. et al. Nat Commun 2020, 11, 4195, under a CC BY 4.0 license [74].

### 3.2. Neuromodulation

#### 3.2.1. Electrical Stimulation

Non-invasive transcranial direct current stimulation (tDCS)/transcranial alternating current stimulation (tACS): In transcranial electrical stimulation, the impedance and charge-transfer stability of the scalp–electrode interface determine the reproducibility of the stimulation dose. FallahRad, Bikson et al. developed a single-use wearable electrotherapy platform entirely free of electronic components (Figure 4A). Composed only of a printed zinc–manganese dioxide battery pack and ionically conductive hydrogel electrodes, this device delivers a self-limited discharge that automatically starts and stops upon skin contact; the authors validated this principle in a bifrontal tDCS montage (2.5 ± 1 mA), with current-flow modeling predicting an electric field of up to 2.3 V/m at the dorsolateral prefrontal cortex and tolerability confirmed in human trials [75].

For wearable applications spanning the body surface beyond the scalp, an approach that raises the hydrogel’s own mixed ionic–electronic conductivity to lower impedance across the transcutaneous electrical nerve stimulation (TENS) frequency band has also been reported. Hwang et al. developed a full-body electrical stimulation suit integrating a dual-conductive hydrogel (DMCH) electrode—combining PEDOT:PSS and LiCl—into a textile compression garment. This hydrogel electrode achieved lower impedance and higher current density than a purely ionic hydrogel across the typical TENS frequency range (1–150 Hz), while integrated resistive pressure sensors on the forearm detected localized pressure variations and adjusted stimulation intensity in real time to deliver personalized tactile feedback (Figure 4B). A 24 h skin-contact test showed stable biocompatibility with no adverse response, demonstrating that haptic feedback and therapeutic neuromuscular stimulation could be integrated into a single wearable platform [76].

Invasive neural stimulation: In invasive neural stimulation, stimulation efficiency and safety are jointly determined by how conformally the gap between electrode and neural tissue is filled, and by whether a chronically stable, low-impedance interface is maintained after insertion. Liu et al. fabricated an elastic thin-film microelectrode array using a highly conductive soft hydrogel as the core. Owing to the hydrogel’s high volumetric capacitance, 20 μm electrode features achieved a current-injection density roughly 30 times higher than platinum electrodes of the same area, enabling localized, low-voltage stimulation of the sciatic nerve in live mice [65]. An injectable hydrogel (ICAA) developed by Yang et al. filled, by injection alone, the gap between a large commercial cuff electrode and a fine vagus nerve, achieving conformal contact and a stable, low impedance; this enabled safe, long-term chronic vagus nerve stimulation for the treatment of myocardial infarction [77].

Implantable interfaces are also being extended beyond direct nerve stimulation toward converting sensed information back into neural or muscular stimulation signals. Kang et al. incorporated a fully implantable wireless tactile sensory system (WTSA), which replaces damaged tactile nerves, into a collagen–fibrin-based artificial skin. The hydrogel surrounding the neural interface electrode exhibited favorable compressive stress–strain behavior and was found to markedly lower the electrochemical impedance relative to a bare Au electrode, reaching a level comparable to that of an Au-blacked Au electrode (Figure 4C), supporting the notion that the hydrogel coating provided both mechanical compliance and low interfacial impedance while minimizing the foreign body response. This enabled electrical stimulation to be delivered to the leg with intensity proportional to the sensed pressure signal, with the resulting leg-movement angle and electromyographic (EMG) response quantitatively verifying this proportional relationship, demonstrating that skin regeneration and tactile function restoration could be achieved simultaneously through a single implantable interface [78]. In contrast to these injectable and coating-based approaches, Wang, F. et al. presented a method of shaping PEDOT:PSS-based inks via direct-ink-writing (DIW) 3D printing to print multi-electrode arrays directly onto the surface of the heart. With a tissue-like Young’s modulus of 650 kPa and an interfacial toughness of 200 J/m^2^, this printed electrode array was able to spatially map epicardial electrophysiological signals in both normal and arrhythmic rat heart models, and its ability to deliver electrical stimulation to the lesion site was separately validated in an acute myocardial infarction model [79].

#### 3.2.2. Ultrasound-Based Non-Invasive Stimulation (Acoustic Impedance Matching)

Because ultrasound neuromodulation must transmit energy through the skull or skin, how well the acoustic impedance is matched between the transducer and the tissue governs stimulation efficiency. This matching has conventionally been handled by gel-based couplants, which are prone to dehydration and thus poorly suited to long-term wear; to overcome this, adhesive hydrogel-based couplants have recently been introduced.

Lee et al. proposed a calcium-modified silk patch with an acoustic impedance of 2.15 × 10^6^ Pa·s·m^−1^ (2.15 MRayl), close to that of human skin (1.99 × 10^6^ Pa·s·m^−1^; 1.99 MRayl), reducing ultrasound transmission loss to approximately 0.002 dB, and confirmed that this impedance-matching approach could also be applied to transcranial ultrasound stimulation [80]. Building on this same principle, Tang, K.W.K. et al. developed a wearable ultrasound device comparable in size to a standard electrophysiological electrode, integrating a bioadhesive hydrogel as the coupler to enable long-term ultrasound stimulation of the somatosensory cortex (Figure 4D). Their Fresnel-lens-based self-focusing transducer achieved an acoustic intensity of 30.7 W/cm^2^ and a peak pressure of 1.92 MPa with a 10 mm focal depth, while the hydrogel couplant exhibited less than 13% acoustic attenuation and maintained a stable adhesion force of 0.961 N/cm for 35 days. Using this system, the authors successfully suppressed somatosensory evoked potentials elicited by functional electrical stimulation over 28 days, demonstrating the platform’s potential for long-term, wearable ultrasound neuromodulation [81]. Meanwhile, the bioadhesive hydrogel–elastomer hybrid couplant developed by Wang, C. et al. (Zhao group) kept a rigid piezoelectric probe stably attached to the skin for 48 h, enabling continuous imaging of deep internal organs; although developed for imaging rather than stimulation, this suggests that the same hydrogel-coupling principle can be applied in common to both diagnostic (imaging) and therapeutic (stimulation) applications [82].

**Figure 4:**
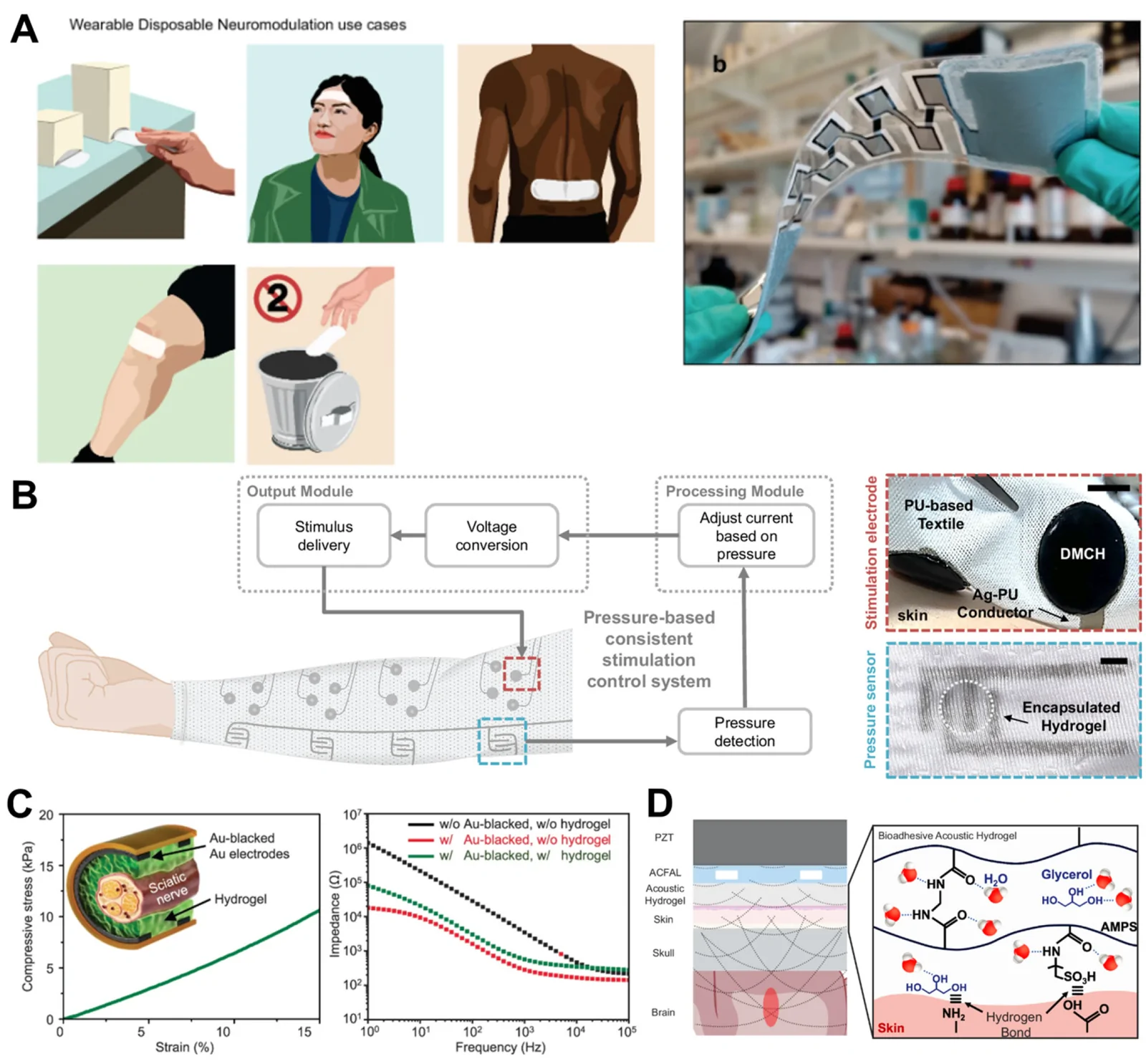
Representative wearable hydrogel-based platforms for electrical and haptic neurostimulation. (**A**) General platform concept of application-specific, single-use wearable electrotherapy devices distributed and used akin to disposable bandages or pharmaceuticals—spanning applications from brain/cranial nerve stimulation (e.g., cognition, headache) to pain relief and wound healing—together with a photograph of an exemplary device; the bi-frontal tDCS configuration discussed in the text is one specific implementation of this platform Adapted from FallahRad, M. et al. Nat Commun 2025, 16, 9060, under a CC BY-NC-ND 4.0 license. © 2025 The Author(s) (The lowercase “b” visible in the right-hand image is retained from the original source figure and does not denote a separate subpanel in the present figure.) [75]. (**B**) Flowchart illustrating the real-time haptic feedback mechanism on the forearm, in which integrated resistive pressure sensors detect localized pressure variations and adjust stimulation intensity accordingly to provide personalized tactile feedback. Adapted from Hwang, J.H. et al. Nat Commun 2026, 17, 4764, under a CC BY 4.0 license [76]. (**C**) Compressive stress–strain curve of the hydrogel, and electrochemical impedance spectra comparing a bare Au electrode, an Au-blacked Au electrode, and an Au-blacked Au electrode coated with hydrogel. Adapted from Kang, K. et al. Nat Commun 2024, 15, 10, under a CC BY 4.0 license [78]. (**D**) Side-view schematic of the layered bioadhesive hydrogel structure, showing its chemical composition and adhesion mechanisms. Adapted from Tang, K.W.K. et al. Nat Commun 2025, 16, 4940, under a CC BY-NC-ND 4.0 license. © 2025 The Author(s) [81].

### 3.3. Device Control

Hydrogels possess excellent biocompatibility and tissue-like mechanical properties, making them a key material for mediating physical human–robot interaction (pHRI). As these transmit high-quality electrophysiological signals with low interfacial impedance while forming a soft, stable physical bond with skin and tissue, this can be viewed as an extension of the impedance-reduction strategies discussed in Section 2.3 into the applied domain of actual robot control. Beyond simple biosignal acquisition, hydrogels have recently evolved into intelligent interfaces that enable real-time control of robotic prostheses, exoskeletons, and mobile robots, as well as the delivery of tactile feedback. The following discussion organizes hydrogel-based interfaces into two axes, wearable and implantable/injectable, and examines specific robotic application cases.

#### 3.3.1. Wearable Hydrogel-Based Robot Control

Wearable hydrogel electrodes acquire high-precision surface electromyography (sEMG) and strain signals from the skin surface, enabling intuitive control of robotic hands and prostheses. Lai et al. developed a 3D-printed polyion complex glycerol (PIC-G) hydrogel electrode that forms smooth, conformal contact with the skin (Figure 5A). This electrode classified 26 hand gestures with 87.4% accuracy and successfully achieved real-time control in which a prosthetic hand reproduced the volunteer’s set of hand gestures (Figure 5B) [83]. Yu, Z. et al. combined a PCGK-CT hydrogel electrode, which features self-healing and excellent self-adhesive properties, with a PSO-ELM algorithm to control a biomimetic robotic hand with multiple degrees of freedom at 94.4% accuracy [84]. Azizi et al. achieved ultralow electrical hysteresis using a hydrogel, allowing a robotic hand to mimic human gestures in real time without delay [85]. Liu et al. utilized a gelatin-based PGBL hydrogel sensor to digitize the bending of fingers and joints in real time, implementing the synchronized control of a robotic hand [86].

Beyond robotic hand control, hydrogel interfaces have also expanded into bi-directional interactions that relay the robot’s sensed information back to the user. Cheng et al. designed a robotic electronic skin (RES) integrating a piezoresistive strain sensor on the back of the robotic finger and a capacitive pressure sensor on the fingertip, improving object-grasping capability in a self-adaptive manner via negative force feedback control [87]. Fang et al. developed a bi-directional HMI system connecting a multi-modal sensory exoskeleton glove and a smart robotic hand using a starch-based hydrogel. When the robotic hand perceives the hardness and shape of an object, the user receives tactile feedback through vibration motors in the exoskeleton glove, alongside visual information [88].

Wearable hydrogels have also expanded into exoskeleton and mobile robots. Wang et al. applied an EMG–FMG fused hydrogel interface to an active rehabilitation robotic glove, demonstrating improved motion-intention decoding accuracy compared to a single EMG channel; based on this, they developed a system to detect the subtle active motor intentions of stroke patients and assist rehabilitation training [89]. Li, M. et al. integrated a CPSD hydrogel sensor with a lower-limb rehabilitation exoskeleton system to reliably acquire EMG and strain signals even during dynamic walking, thereby identifying the user’s gait intentions [90].

The use of hydrogel electrodes is not limited to myoelectric signals; it has also expanded to other electrophysiological signals such as electrooculography (EOG), broadening the input channels available for robot control. Chowdhury et al. attached PVA-based hydrogel bioelectrodes around the eyes to collect high-quality EOG signals and controlled an unmanned drone by mapping changes in the user’s gaze direction onto its flight trajectory using a real-time algorithm [91].

#### 3.3.2. Implantable Hydrogel-Based Robot Control

Although numerous prior studies have used wearable hydrogels as interfaces to control robots, cases directly applying implantable hydrogels to robotic applications remain rare. This suggests that while wearable hydrogels have already matured as sEMG electrodes, the application of implantable hydrogels to robot control is still at an early stage. Jin et al. developed a conductive hydrogel (IT-IC hydrogel) combining phenylboronic acid (PB)-mediated multiple crosslinking with in situ reduced gold nanoparticles (AuNPs) as an injectable tissue prosthesis. This hydrogel is injected into irregular gaps in muscle and nerve tissue, relieving the shear stress generated during injection while instantaneously restoring bidirectional electrical signal transmission both between tissues and between tissue and device. The researchers injected this hydrogel into animals with volumetric muscle loss (VML) and peripheral nerve injury, and constructed a robot-assisted closed-loop rehabilitation (C-RAR) system by interfacing it with a stretchable bioelectronic device (SH-SB patch). In this compliant control system, stimulation delivered to the peripheral nerve elicits an EMG signal in the muscle, which is monitored via an epimysial hydrogel electrode; when the collected signal exceeds a target threshold, the exoskeleton robot immediately lifts the affected leg to assist walking. This enabled immediate gait rehabilitation with robotic assistance even at an early stage of severe tissue damage, and the system also demonstrated clinical utility by restoring the electrophysiological environment to accelerate long-term muscle fiber regeneration [92,93].

## 4. Conclusions and Future Perspectives

Over the past decade, the field of neural interfaces has advanced steadily toward overcoming the fundamental limitations of conventional metal- and silicon-based electrodes. Two obstacles have proven especially persistent: the chronic foreign body response (FBR) driven by the modulus mismatch between rigid electrodes and soft tissue, and the high interfacial impedance that arises when electronically conductive materials meet ionically conductive tissue. The functional hydrogels reviewed here address both through a tissue-like modulus and a diversified set of conduction mechanisms spanning ionic, electronic, and nanocomposite pathways. Critically, these materials have progressed beyond property-level validation (Section 2) into functioning systems (Section 3)—encompassing neural recording, electrical and ultrasound stimulation, and robotic control. Across these applications, one design principle recurred: the conformal-contact strategy, in which a liquid precursor is delivered and gelled in situ to bond gaplessly to complex tissue surfaces, emerged as a factor governing interfacial performance at least as decisive as the choice of conduction mechanism itself.

For these advances to move beyond laboratory-scale proof of concept toward clinical use and practical robotic systems, however, several interlocking challenges must first be resolved.

(1) Precise control of gelation kinetics: The gelation strategies reported for injectable and implantable precursors—spontaneous phase separation, thermally induced phase transition, and reaction-rate-modulated crosslinking, among others—remain sensitive to environmental conditions and body-temperature fluctuations. Regardless of the specific chemistry, this sensitivity makes it difficult to reliably reproduce the narrow “Goldilocks” window in which the material stays deliverable as a liquid yet resists premature flow. Designs that incorporate stimuli-responsive chemistry to actively gate the timing of gelation will be needed.
(2) Long-term in vivo stability and predictable degradation: Most studies of implantable hydrogels report only short-term performance over a few weeks, leaving a gap in long-term data on interfacial stability, degradation, and property loss after implantation. Swelling deserves particular attention in this context: dimensional and hydration changes that develop over weeks alter both the mechanical footprint and the interfacial impedance of an implanted hydrogel, yet the swelling ratio and its time course are rarely reported alongside the electrical metrics that are. Degradable crosslinking chemistries whose degradation timing can be quantitatively tuned, validated in chronic animal models, will be required to close this gap.
(3) Structural integration with robotic hardware: Wearable hydrogel sensors remain largely confined to standalone patch formats that interface with the skin independently of the surrounding hardware. When worn with an exoskeleton or robotic suit, this introduces the inconvenience of double-donning, as well as slippage artifacts from relative body motion. What is needed are system-level designs in which the sensor is structurally integrated with the skin on one side and with the garment or exoskeleton frame on the other, rather than treated as a separate add-on.
(4) Clinical indications for implantable systems: Implantable hydrogel-based control has so far been demonstrated only in a narrow set of severe conditions such as volumetric muscle loss. Work is needed to define the distinct therapeutic advantages of implantable approaches over non-invasive wearable technologies, and to identify the clinical indications for which they are best suited.

These challenges are not independent but systemic and mutually dependent. Precise control of gelation kinetics is a prerequisite for predictable degradation; the long-term stability this enables is, in turn, what makes structural integration with robotic hardware and the expansion of implantable indications meaningful to pursue. Addressed together through a full-cycle approach spanning material design, system integration, and clinical translation, hydrogels are poised to establish themselves not merely as biomaterials but as a core platform for next-generation neural interfaces and bioelectronic robotic systems.

## Figures and Tables

**Figure 5:**
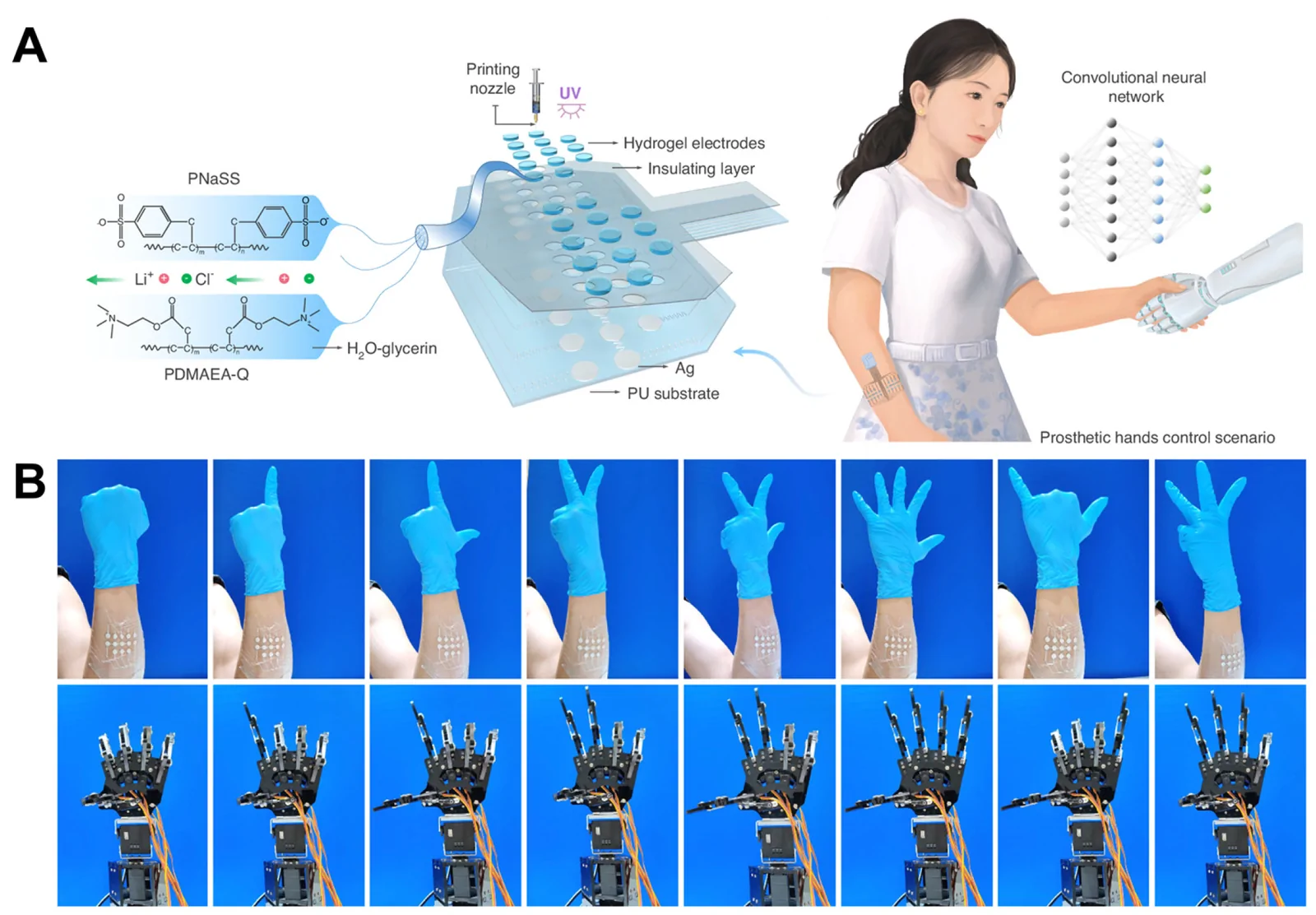
Hydrogel-based sEMG electrode system for wearable prosthetic hand control. (**A**) Schematic illustration of the PIC-G gel structure, the layer-by-layer construction of the sEMG electrode array, and a conceptual demonstration of the sEMG patch for smart control of a prosthetic hand. (**B**) Photographs showing a prosthetic hand executing a set of hand gestures via the system. Adapted from Lai, J. et al. Microsyst. Nanoeng. 2025, 11, 15, under a CC BY 4.0 license [83].

**Table 1:** Comparison of representative conduction strategies in functional hydrogels for neural interfacing: materials and compositions, measured conductivities reported in a common unit (S/cm), and the underlying mechanisms. Original mechanisms, advantages, and limitations are retained, with network, mechanical, and characterization data added for material-level comparison.

Conduction Type	Strategy Network/Crosslinking	Material and Composition Mechanical Properties	Conductivity (S/cm) Transport Mode	Mechanism, Advantages and Limitations, Characterization	**Refs**
(1) Ionic conduction	Post-gelation ion infiltration (immersion). Network: reconstructed ionic alginate; physical PVA/HPC; cellulose–bentonite coordination.	Pre-crosslinked network immersed in an ionic bath: Ca^2+^/Fe^3+^- or Al^3+^-crosslinked alginate loaded with Li^+^ [52]; HPC/PVA (0–3.75 wt% HPC) in 1–5 m NaCl [53]; cellulose–bentonite in 2 M LiCl [55]. Mechanical: alginate E = 0.46–1.2 GPa; strength ≤ 55 MPa [52]. HPC/PVA: stress ≤ 1.3 MPa; strain ≤ 975% [53]. Cellulose–bentonite: fracture stress 0.76 MPa; strain 96% [55].	2.2 × 10^−3^ and 7.8 × 10^−3^ (Ca^2+^/Fe^3+^- and Al^3+^-crosslinked alginate–Li) [52]; 1.7 × 10^−2^– 3.4 × 10^−2^ (HPC/PVA, 5 m NaCl, 1 MHz) [53]; 8.99 × 10^−2^ (cellulose–bentonite, 2 M LiCl; ≈90× the same network without LiCl) [55]. Mode: ionic.	- Mechanism: ion diffusion through a 5–50 nm mesh. - Advantages: conductivity tunable after gelation without altering the network; carriers match those of tissue. - Limitations: set by the bath rather than by the material; conductivity vanishes once the ions are removed [52]. - Characterization: tensile/compression; SEM; XRD/FTIR/XPS; conductivity.	[52,53,54,55]
	Direct ion doping of the precursor. Network: salt/ionic-liquid incorporation; visible-light photo crosslinking [57]; ionic + thiolene double network [58].	Concentrated salt or ionic-liquid monomer blended into the precursor before gelation: alginate–polyacrylamide with 5 wt% LiCl [56]; bio-ionic-liquid-conjugated network [57]; click-ionogel [58]; salt-containing polyacrylamide networks [59,60,61]. Mechanical: compressive E: Gel-MA/Bio-IL 0.60–32.07 kPa; PEG-DA/Bio-IL 1.27–178.13 kPa [57]. Click-ionogel: stress 2.28 MPa at 1390% strain [58].	10^−5^–10^0^ across salt-doped polyacrylamide networks [59,60,61]. Mode: ionic.	- Mechanism: raised carrier concentration(270 Ω → MΩ range without the LiCl) [56]. - Advantages: single-step; no post-treatment required. - Limitations: dopant free to leach in vivo; upper concentration bounded by osmotic balance. - Characterization: conductivity; swelling; tensile/compression; rheology.	[56,57,58,59,60,61]
(2) Electronic and mixed ionic–electronic conduction (conducting-polymer matrix)	Bicontinuous phase-morphology control. Network: bicontinuous PEDOT:PSS/hydrophilic-PU phases.	PEDOT:PSS blended with hydrophilic polyurethane at a tuned mass ratio. Mechanical: stretchability > 400%; fracture toughness > 3300 J/m^2^ [20].	>11. Mode: mixed ionic–electronic.	- Mechanism: percolating PEDOT-rich phase, continuous π-electron pathway. - Advantages: simple blending, no post-treatment. - Limitations: lowest conductivity among the conducting-polymer examples listed here. - Characterization: conductivity; tensile/fracture tests; morphology.	[20]
	Template-directed fiber assembly. Network: crosslinked PAA template with H-bonded PEDOT:PSS fibers.	Ultrathin PEDOT:PSS fiber network grown along poly(acrylic acid) (PAA) chains. Mechanical: E = 25 kPa; strain at break 610%; toughness 1 MJ/m^3^ [63].	247. Mode: mixed ionic–electronic.	- Mechanism: template-guided fiber interconnection, continuous conducting-polymer network. - Advantages: high conductivity retained in a soft matrix. - Limitations: requires a second, template network. - Characterization: conductivity; tensile/compression; microstructure.	[63]
	Secondary doping/chain reorganization. Network: PEDOT/PSS reorganization into interconnected PEDOT-rich pathways.	Pure PEDOT:PSS hydrogel treated with sulfuric acid or a strongly ionic liquid. Mechanical: E = 32 ± 5.1 kPa (compression); 24 ± 5.4 kPa (AFM) [65].	47.4. Mode: mixed ionic–electronic.	- Mechanism: PEDOT^+^–PSS^−^ dissociation, enhanced π–π stacking. - Advantages: no additive remains in the final gel. - Limitations: requires harsh acid or ionic-liquid treatment. - Characterization: compression/AFM; EIS; spectroscopy/morphology.	[64,65]
	Laser-induced phase separation. Network: laser-induced interconnected PEDOT-rich domains.	Pure PEDOT:PSS hydrogel, laser-processed. Mech.: not reported [62].	670. Mode: mixed ionic–electronic.	- Mechanism: local formation of a PEDOT-rich continuous phase. - Advantages: spatially selective patterning of conductive regions. - Limitations: additional processing step and equipment. - Characterization: microscopy/phase analysis; conductivity; stability.	[62]
(3) Filler-based nanocomposite	Metallic-filler hybridization. Network: Ag-flake percolation in partially dehydrated PAAm–alginate.	Micron-scale Ag flakes percolating in a PAAm–alginate matrix. Mechanical: E < 10 kPa [66].	374. Mode: electronic/percolative.	- Mechanism: charge transport across contacting metal flakes. - Advantages: highest conductivity among the filler-based examples listed here. - Limitations: stiffening scales with filler loading. - Characterization: conductivity; deformation; microstructure.	[66]
	Percolation with mechanically induced filler alignment. Network: semicrystalline PVA with cyclically aligned CNT pathways.	High-aspect-ratio CNTs in a semicrystalline PVA matrix, cyclically stretched (20% strain, up to 5000 cycles). Mechanical: strain at break 64.5 ± 7.9%; fatigue > 20,000 cycles at 20% strain [68].	Bulk conductivity not reported; interfacial impedance 33.20 ± 9.27 kΩ at 1 kHz [68] Mode: electronic/percolative; conductivity not reported.	- Mechanism: shortened path along the alignment axis, direction-selective impedance drops. - Advantages: directional conductivity gain. - Limitations: requires mechanical training; vulnerable to agglomeration and filler migration. - Characterization: EIS; cyclic tensile tests; electron/Raman microscopy; X-ray.	[11,68,70]

All conductivity values are reported in S/cm. For reference, the intrinsic ionic conductivity of biological tissue is 3 × 10^−3^–7 × 10^−3^ S/cm (0.3–0.7 S/m) [17]. Young’s moduli: immersion-type ionic hydrogels 0.59 MPa (HPC/PVA) [53] to 0.46–1.2 GPa (reconstructed alginate) [52]; conducting-polymer hydrogels (type 2) 100 kPa–1 GPa [11,62]; filler-based nanocomposites (type 3) 1 kPa–10 MPa, with conductivities spanning 10^−5^–10^2^ S/cm as a class [11,70]. Impedance values are reported in the Mechanism, advantages and limitations column so that they are never combined with conductivity in a single cell. Mechanical values are included only when reported in the cited studies and should be interpreted under the original test conditions; NR denotes not reported. Table-specific abbreviations: HPC, hydroxypropyl cellulose; gelatin methacryloyl (GelMA), gelatin methacryloyl; Bio-IL, bio-ionic liquid; PEGDA, poly(ethylene glycol) diacrylate; SEM, scanning electron microscopy; XRD, X-ray diffraction; FTIR, Fourier-transform infrared spectroscopy; XPS, X-ray photoelectron spectroscopy; AFM, atomic force microscopy; EIS, electrochemical impedance spectroscopy; E, elastic modulus.

## Data Availability

No new data were created or analyzed in this study. Data sharing is not applicable to this article.

## Abbreviations

The following abbreviations are used in this manuscript:

**Abbreviation**	**Definition**
CNT	carbon nanotube
DMSO	dimethyl sulfoxide
ECG	electrocardiography
ECM	extracellular matrix
EEG	electroencephalography
EMG	electromyography
FBR	foreign body response
GelMA	gelatin methacryloyl
GSH	glutathione
MTT	3-(4,5-dimethylthiazol-2-yl)-2,5-diphenyltetrazolium bromide
PAA	poly(acrylic acid)
PAAm	polyacrylamide
PANI	polyaniline
PDMS	polydimethylsiloxane
PEDOT:PSS	poly(3,4-ethylenedioxythiophene):poly(styrene sulfonate)
PEG	poly(ethylene glycol)
PET	poly(ethylene terephthalate)
PETRA	pentaerythritol triacrylate
PPy	polypyrrole
PVA	poly(vinyl alcohol)
SNR	signal-to-noise ratio
tACS	transcranial alternating current stimulation
tDCS	transcranial direct current stimulation
TENS	transcutaneous electrical nerve stimulation
